# A Synthetic Triterpenoid CDDO-Im Inhibits Tumorsphere Formation by Regulating Stem Cell Signaling Pathways in Triple-Negative Breast Cancer

**DOI:** 10.1371/journal.pone.0107616

**Published:** 2014-09-17

**Authors:** Jae Young So, Janice J. Lin, Joseph Wahler, Karen T. Liby, Michael B. Sporn, Nanjoo Suh

**Affiliations:** 1 Department of Chemical Biology, Ernest Mario School of Pharmacy, Rutgers, The State University of New Jersey, Piscataway, New Jersey, United States of America; 2 Department of Pharmacology, Dartmouth Medical School, Hanover, New Hampshire, United States of America; 3 Rutgers Cancer Institute of New Jersey, New Brunswick, New Jersey, United States of America; University of Alabama at Birmingham, United States of America

## Abstract

Triple-negative breast cancer is associated with poor prognosis because of a high rate of tumor recurrence and metastasis. Previous studies demonstrated that the synthetic triterpenoid, CDDO-Imidazolide (CDDO-Im) induced cell cycle arrest and apoptosis in triple-negative breast cancer. Since a small subpopulation of cancer stem cells has been suggested to be responsible for drug resistance and metastasis of tumors, our present study determined whether the effects of CDDO-Im in triple-negative breast cancer are due to the inhibition of a cancer stem cell subpopulation. CDDO-Im treatment markedly induced cell cycle arrest at G2/M-phase and apoptosis in the triple-negative breast cancer cell lines, SUM159 and MDA-MB-231. Because SUM159 cells were more sensitive to CDDO-Im than MDA-MB-231 cells, the effects of CDDO-Im on the cancer stem cell subpopulation were further investigated in SUM159 cells. SUM159 cells formed tumorspheres in culture, and the cancer stem cell subpopulation, CD24−/EpCAM+ cells, was markedly enriched in SUM159 tumorspheres. The CD24−/EpCAM+ cells in SUM159 tumorspheres were significantly inhibited by CDDO-Im treatment. CDDO-Im also significantly decreased sphere forming efficiency and tumorsphere size in both primary and secondary sphere cultures. PCR array of stem cell signaling genes showed that expression levels of many key molecules in the stem cell signaling pathways, such as Notch, TGF-β/Smad, Hedgehog and Wnt, were significantly down-regulated by CDDO-Im in SUM159 tumorspheres. Protein levels of Notch receptors (c-Notch1, Notch1 and Notch3), TGF-β/Smad (pSmad2/3) and Hedgehog downstream effectors (GLI1) also were markedly reduced by CDDO-Im. In conclusion, the present study demonstrates that the synthetic triterpenoid, CDDO-Im, is a potent anti-cancer agent against triple-negative breast cancer cells by targeting the cancer stem cell subpopulation.

## Introduction

Triple-negative breast cancer is a subtype of breast cancer, defined by the lack of estrogen receptors, progesterone receptors and human epidermal growth factor receptor 2 (HER2) [1]. The triple-negative subtype accounts for 12% to 24% of human breast cancers and is associated with a significantly higher rate of relapse and lower overall survival rate than other breast cancer subtypes [1]. Despite the high sensitivity of triple-negative breast cancer to initial chemotherapy, the high rate of early recurrence and the absence of targeted therapies have been major challenges to treat these patients [2]. Approximately 20% of triple-negative breast patients carry *BRCA* mutations; thus drugs affecting the DNA repair system, such as platinum compounds and poly ADP ribose polymerase (PARP) inhibitors, have been investigated as potential therapies [3]. However, the other 80% of triple-negative breast cancer patients without *BRCA* mutations might not benefit from those therapies, requiring the development of new therapeutic agents [3].

Cancer stem cells (also known as tumor-initiating cells) are the subpopulation of cancer cells shown to be required for sustained tumor growth and progression as well as for tumor recurrence and metastasis [4]. In breast cancer, these cancer stem cells are enriched as a subpopulation of cells with CD44^+^/CD24^−/low^ phenotype and form tumors in animals with as few as 100 cells [4]. Other studies showed that CD44^+^/CD24^−/low^ cells are resistant to chemotherapy and radiotherapy [5], [6], [7]. Moreover, the CD44^+^/CD24^−/low^ cells are more abundant in triple-negative breast cancer than in other subtypes [8], [9], suggesting that the cancer stem cells are a source of tumor relapse. Interestingly, many of the signaling pathways that regulate normal stem cells, such as Wnt, Hedgehog and Notch, are aberrantly activated in cancer stem cells [10], [11], [12]. Since the activation of stem cell signaling pathways is required for the maintenance of these cells, new experimental agents inhibiting these pathways are being developed to target cancer stem cells [13].

CDDO, 2-cyano-3,12-dioxooleana-1,9(11)-dien-28-oic acid, is a synthetic triterpenoid derived from the naturally occurring triterpene oleanolic acid [14], [15]. To further increase its anti-cancer and anti-inflammatory properties, numerous derivatives of CDDO, such as CDDO-methyl ester (CDDO-ME), CDDO-ethyl amide (CDDO-EA) and CDDO-imidazolide (CDDO-Im), were developed [15]. CDDO-Im is one of the most potent synthetic triterpenoids shown to induce growth inhibition and apoptosis in various human cancer cells, including multiple myeloma, lung, pancreas and breast cancer [16], [17], [18], [19], [20]. In breast cancer, CDDO-Im is effective on both ER-positive and ER-negative breast cancer cells [16], [21]. Development of mammary tumors in the HER2-overexpressing animal model was delayed by CDDO-Im [20]. A recent study also demonstrated that CDDO-Im induced apoptosis in BRCA1-deficient breast cancer cells by increasing DNA damage and G2/M arrest [19]. In the present study, we investigated the effect of CDDO-Im on the cancer stem cell subpopulation in triple-negative breast cancer cells. Multiple stem cell signaling pathways were examined as potential targets of CDDO-Im to inhibit the cancer stem cells in triple-negative breast cancer.

## Materials and Methods

### Reagents and cell culture

**Figure 1:**
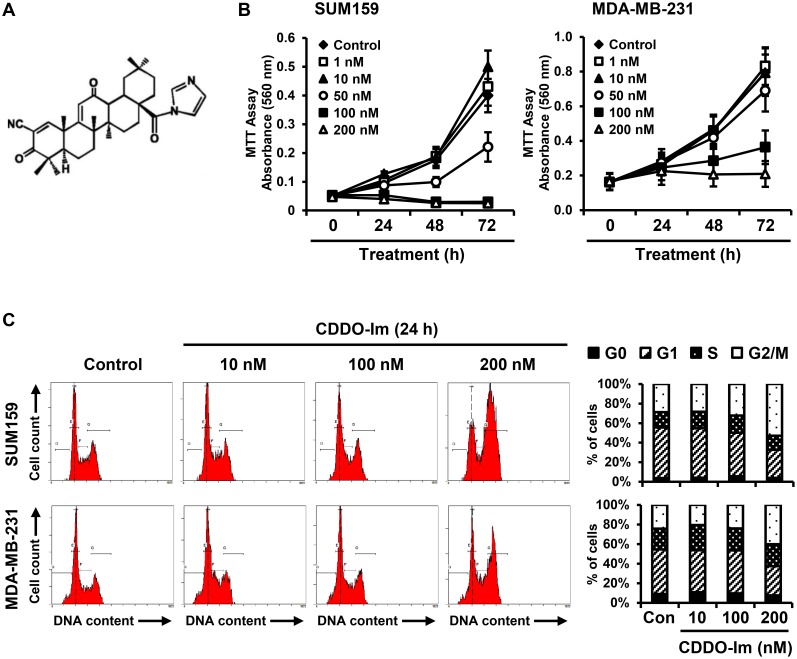
Induction of G2/M phase arrest by CDDO-Im inhibits the growth of triple-negative breast cancer cells. (**A**) The structure of 1-[2-Cyano-3,12-dioxooleana-1,9(11)-dien-28-oyl]-imidazole (CDDO-Im). (**B**) SUM159 and MDA-MB-231 cells were incubated with vehicle control or CDDO-Im (1, 10, 50, 100 or 200 nM), and cell proliferation was measured by an MTT assay at 0, 24, 48 and 72 h time points. Three separate experiments in quadruplicate were conducted, and averages of the results are shown. (**C**) SUM159 and MDA-MB-231 cells were incubated with vehicle control or CDDO-Im (10, 100 or 200 nM) for 24 h, and the cell cycle distribution (G0, G1, S and G2/M phase) was measured by flow cytometry. Three separate experiments were conducted, and representative results are shown. Averages of the three separate experiments are shown in the graph.

1-[2-Cyano-3,12-dioxooleana-1,9(11)-dien-28-oyl]-imidazole (CDDO-Im) (Fig. 1A) was synthesized as described [22], [23] and dissolved in dimethyl sulfoxide (DMSO; Sigma-Aldrich, St. Louis, MO). SUM159 breast cancer cells, commercially available from Asterand (Detroit, MI), were described previously [24]. SUM159 cells were grown in Ham’s F-12 culture medium supplemented with 5% fetal bovine serum, 1% penicillin/streptomycin, 1 µg/ml hydrocortisone and 5 µg/ml insulin at 37°C and 5% CO_2_. MDA-MB-231 breast cancer cells were from American Type Culture Collection (Manassas, VA) and were grown in DMEM culture medium supplemented with 10% fetal bovine serum and 1% penicillin/streptomycin at 37°C and 5% CO_2_.

### MTT assay

We previously reported the details of the MTT assay [25]. SUM159 and MDA-MB-231 cells were seeded into each well of 96-well plates (1,000 cell/well) and treated the next day with vehicle control or CDDO-Im (1, 10, 50, 100 and 200 nM) for given incubation time. The absorbance was measured with a spectrophotometer (Tecan US, Durham, NC) to determine cell proliferation rate.

### Cell cycle analysis

SUM159 and MDA-MB-231 cells were incubated with vehicle control or CDDO-Im (10, 100 and 200 nM) for 24 h. The cells were then harvested by trypsinization and fixed in ice-cold 70% ethanol for 30 min. The ethanol-fixed cells were incubated with Ribonuclease A solution (1 µg/ml) and PI (1 mg/ml) for 30 min in the dark at room temperature and analyzed by FC500 Analyzer (Beckman Coulter, Brea, CA).

### Apoptosis assay

SUM159 cells were incubated with vehicle control or CDDO-Im (10, 50, 100, 150 or 200 nM) for 24 h. Cells were harvested by trypsinization, and the dead cells in culture medium were also collected by centrifugation. The collected cells were stained with Annexin V and PI using the FITC Annexin V Apoptosis Detection Kit II (BD Pharmigen, San Jose, CA), following the manufacturer’s instruction. The stained cells were analyzed by FC500 Analyzer (Beckman Coulter).

### Tumorsphere culture

SUM159 cells were harvested from monolayer culture using StemPro® Accutase® Cell Dissociation Reagent (Life Technologies, Grand Island, NY) and suspended into complete MammoCult® medium (Stemcell Technologies, Vancouver, Canada). For primary tumorsphere culture, 10,000 cells were seeded into each well of 6-well Corning™ Ultra-Low Attachment Plates (Thermo Fisher Scientific Inc., Waltham, MA) and incubated with vehicle control or CDDO-Im (100 nM) for 7 days without disturbing the plates and without replenishing the medium. For secondary tumorsphere culture, the primary tumorspheres were dissociated into single cells using StemPro® Accutase® Cell Dissociation Reagent (Life Technologies) and filtered through 40 µm Cell Strainer (Thermo Fisher Scientific Inc.) to ensure the single cells suspension. 2,500 cells were seeded into each well of 24-well Corning™ Ultra-Low Attachment Plates (Thermo Fisher Scientific Inc.) and incubated with vehicle control or CDDO-Im (100 nM) for 7 days without disturbing the plates and without replenishing the medium. At the end of each 7-day incubation, the primary and the secondary tumorspheres were gathered at the center of the well by slowly swirling plates, and the pictures were taken to measure number and size of tumorspheres using a TE200 microscope (Nikon Instrument Inc., Melville, NY).

### Flow cytometry

The detailed procedure was previously reported [26]. SUM159 cells were stained with antibodies against EpCAM-FITC (MCA1870F) from AbD Serotec (Raleigh, NC) and CD24-PE-CyTM7 (561646) from BD bioscience (San Jose, CA). The stained cells were then analyzed by FC500 Analyzer (Beckman Coulter) to determine the percentage of subpopulations expressing EpCAM and CD24.

### Western blot analysis

The detailed procedure was described previously [27]. The primary antibody recognizing c-Notch1 (4147), Notch1 (4380), Notch2 (5732), Notch3 (5276), GLI1 (3538), SHH (2207), SUFU (2520), pSmad1/5/8 (9511), pSmad2/3 (8828) and Smad4 (9515) were from Cell Signaling Technology (Beverly, MA); β-Actin was from Sigma-Aldrich. Secondary antibodies were from Santa Cruz Biotechnology (Santa Cruz, CA).

### RT^2^ profiler PCR array

RNA extracted form primary tumorspheres was converted to cDNA and amplified using the RT2 SYBR Green Mastermix and the RT2 First Strand Kit (Qiagen, Valencia, CA), following the manufacturer’s protocol. The expression of 84 genes involved in human stem cell signaling pathways was then analyzed using RT^2^ profiler PCR array with 384-well plate format (Qiagen) as instructed in the manufacturer’s hand book. PCR amplification was conducted by ViiATM 7 real time PCR system (Life Technologies, Carlsbad, CA), and fold change of gene expression was calculated by ddC_t_ methods as previously described [28].

### Statistical analysis

Statistical significance was evaluated using Student’s *t* test.

## Results

### The synthetic triterpenoid, CDDO-Im, induces cell cycle arrest at G2/M-phase and apoptosis in triple-negative breast cancer cells

**Figure 2:**
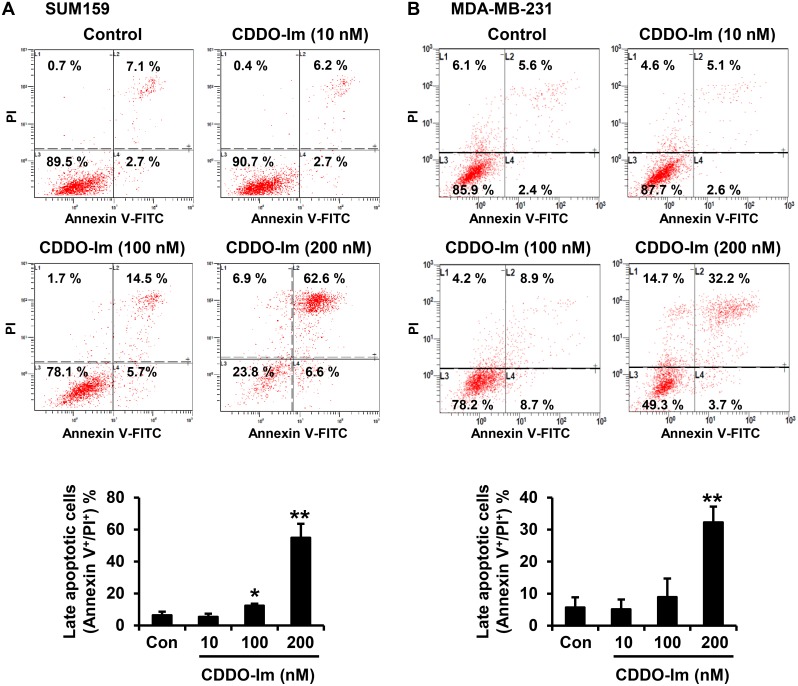
Induction of apoptosis by CDDO-Im in triple-negative breast cancer cells. SUM159 cells (**A**) and MDA-MB-231 cells (**B**) were incubated with vehicle control or CDDO-Im (10, 100 or 200 nM) for 24 h. The cells were stained with annexin V and propidium iodide (PI), and apoptotic cell death was determined by flow cytometry. Three separate experiments were conducted for each cell line, and representative results are shown. Averages of the three separate experiments are shown in the graph (*p<0.05, **p<0.01).

The inhibitory effect of the synthetic triterpenoid, CDDO-Im (Fig. 1A), on the growth of triple-negative breast cancer was investigated with two well-established triple-negative breast cancer cell lines, SUM159 and MDA-MB-231. CDDO-Im treatment at 50, 100 and 200 nM for 72 h significantly inhibited the growth of both SUM159 and MDA-MB-231 cells (p<0.01) (Fig. 1B). CDDO-Im treatment at 200 nM for 24 h markedly induced cell cycle arrest at G2/M-phase in both SUM159 cells (from 28.7±7.5% to 53.1±9.3%, p<0.01) and MDA-MB-231 cells (from 24.3% ±6.3 to 39.8±7.7%, p<0.01) (Fig. 1C). Apoptotic cell death after treatment with CDDO-Im in SUM159 and MDA-MB-231 cells was measured by staining with Annexin V and PI. The percentage of late apoptotic (Annexin V^+^/PI^+^) SUM159 cells was significantly increased by CDDO-Im treatment at 100 and 200 nM for 24 h (Fig. 2A). CDDO-Im treatment at 200 nM for 24 h also significantly induced late apoptotic cell death in MDA-MB-231 cells (Fig. 2B). Although CDDO-Im was effective on both SUM159 and MDA-MB-231 cells, SUM159 cells were more sensitive to CDDO-Im than MDA-MB-231 cells. Therefore, we conducted further studies with SUM159 cells.

### Cancer stem cells (CD24^−^/EpCAM^+^) are enriched with sphere culture and reduced by CDDO-Im treatment in SUM159 triple-negative human breast cancer cells

**Figure 3:**
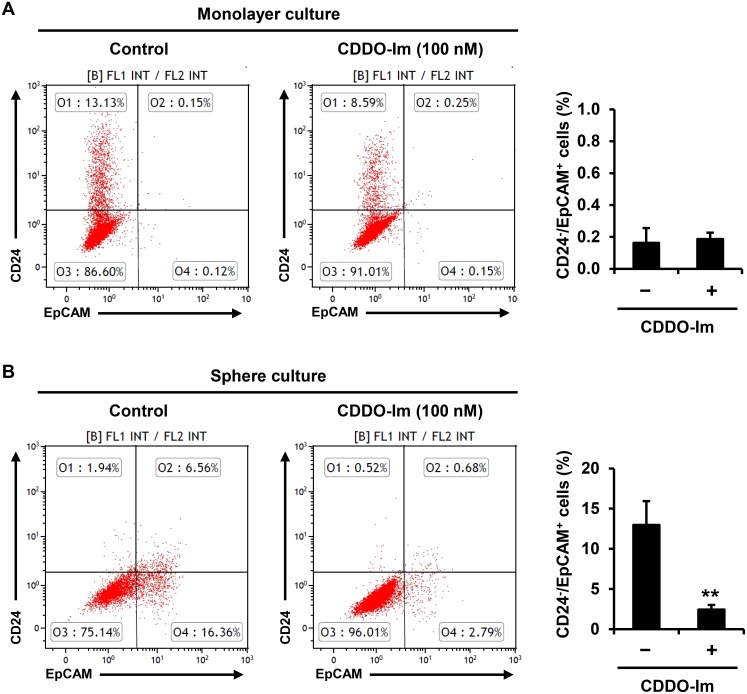
Enrichment of the CD24^−^/EpCAM^+^ subpopulation in tumorspheres of SUM159 cells and its repression by CDDO-Im. (**A**) SUM159 cells were treated with vehicle control or CDDO-Im (100 nM) for 24 h in monolayer culture. Then, SUM159 cells were stained with CD24 and EpCAM, and the number of cells in each subpopulation (CD24^−^/EpCAM^+^, CD24^−^/EpCAM^−^, CD24^+^/EpCAM^+^ and CD24^+^/EpCAM^−^) was determined by flow cytometry. Three separate experiments were conducted, and average percentages of CD24^−^/EpCAM^+^ subpopulation are shown in the graph. (**B**) SUM159 cells were treated with vehicle control or CDDO-Im (100 nM) for 7 days in sphere culture. Then, SUM159 cells were stained with CD24 and EpCAM, and the number of cells in each subpopulation (CD24^−^/EpCAM^+^, CD24^−^/EpCAM^−^, CD24^+^/EpCAM^+^ and CD24^+^/EpCAM^−^) was determined by flow cytometry. Three separate experiments were conducted, and average percentages of CD24^−^/EpCAM^+^ subpopulation are shown in the graph (**p<0.01).

The subpopulation of cells known as cancer stem cells or tumor-initiating cells has been suggested to be responsible for the initiation, progression and recurrence of tumors [29]. In breast cancer, cells with CD44^+^/CD24^−/low^/epithelial cell adhesion molecule (EpCAM)^+^ phenotype were identified as the cancer stem cells [6]. Since more than 99% of SUM159 cells were CD44^+^ cells in both monolayer and sphere cultures [6], we utilized CD24 and EpCAM as additional surface markers to further investigate cancer stem cells. In monolayer culture, the CD24^−^/EpCAM^+^ cancer stem cell subpopulation was less than 1% (0.16±0.09%), and the subpopulation was not significantly changed by CDDO-Im treatment (0.18±0.04%) (Fig. 3A). The CD24^−^/EpCAM^+^ cancer stem cell subpopulation was markedly increased in sphere culture (12.97±2.95%), which was significantly reduced by CDDO-Im treatment (2.44±0.58%) (p<0.001), an 81.2% inhibition (Fig. 3B).

### CDDO-Im decreases sphere forming efficiency and sphere size in both primary and secondary tumorspheres of SUM159 cells

**Figure 4:**
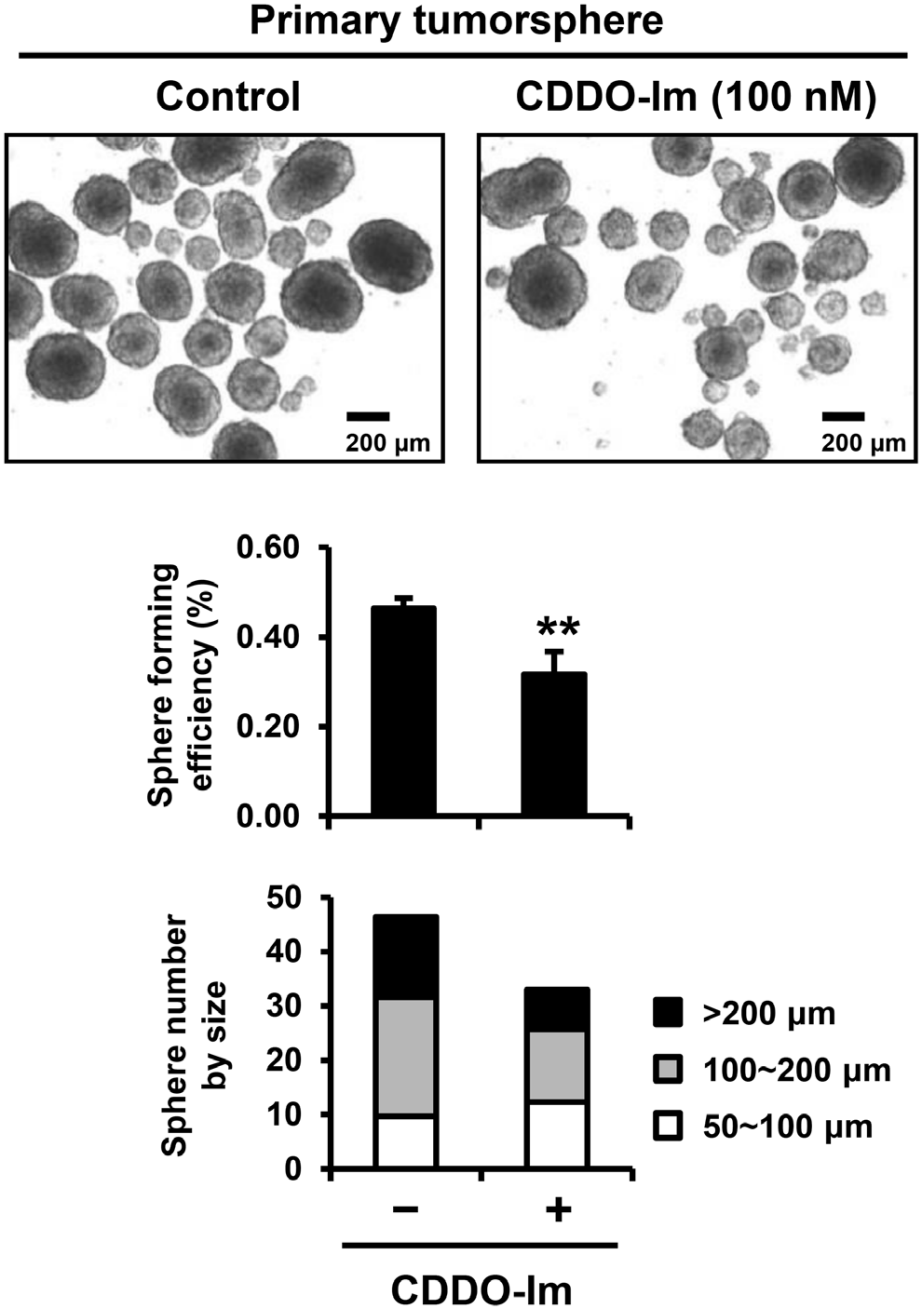
Inhibition of sphere forming efficiency and size of SUM159 tumorspheres by CDDO-Im in primary sphere culture. SUM159 cells were incubated with vehicle control or CDDO-Im (100 nM) in primary sphere culture. Representative microphotographs of tumorspheres after 7-day incubation were shown. Two separate experiments in triplicate were conducted. Averages of the sphere forming efficiency with or without CDDO-Im treatment are shown in the graph (**p<0.01). The size of tumorspheres was divided into three ranges (50∼100, 100∼200 and >200 µm). Average number of tumorspheres in each size range is shown in the graph.

**Figure 5:**
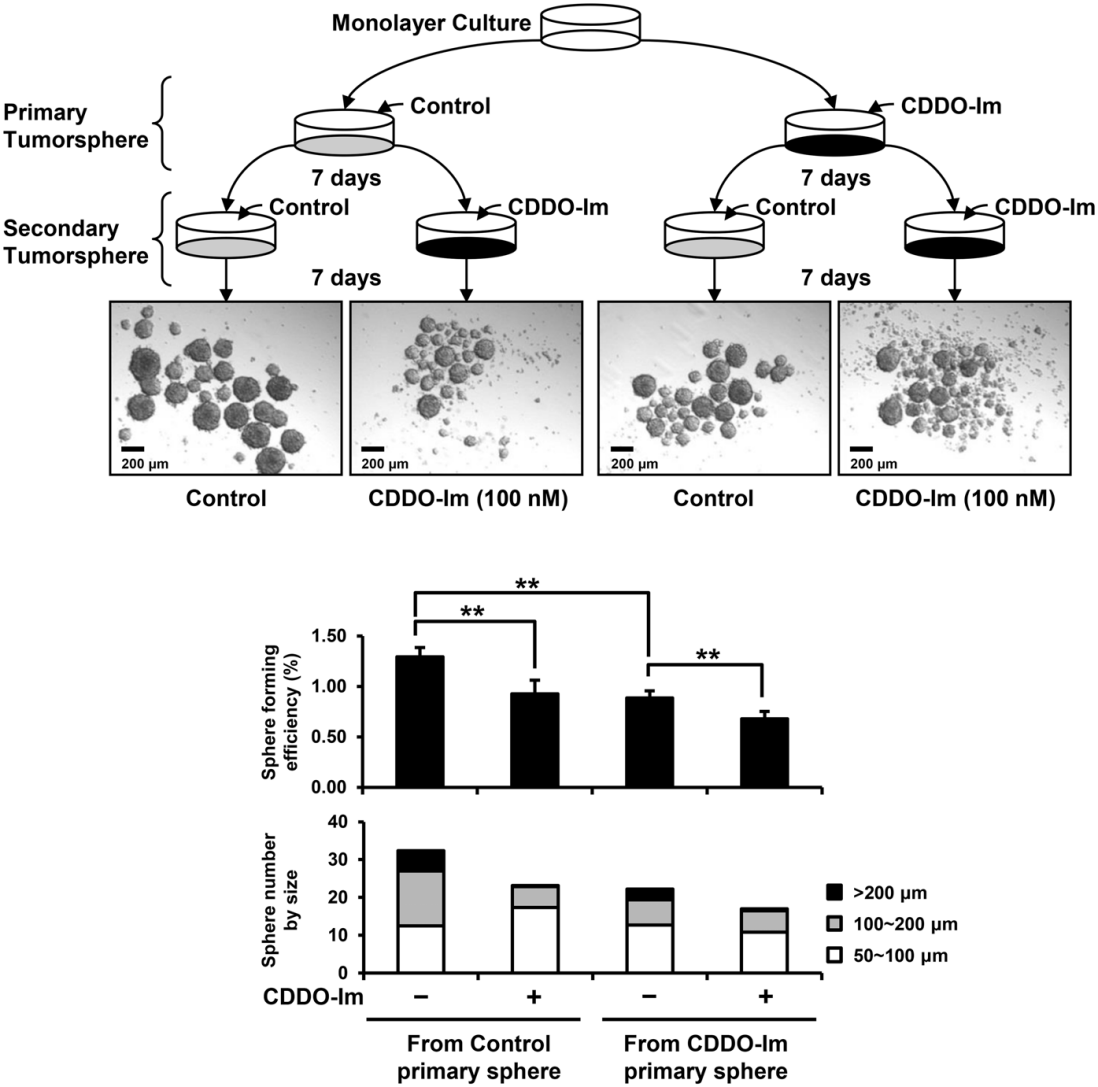
Repression of sphere forming efficiency and size of SUM159 tumorspheres by CDDO-Im in secondary sphere culture. SUM159 cells were harvested separately from primary spheres treated with vehicle control (control primary spheres) or CDDO-Im (CDDO-Im primary spheres). Then the SUM159 cells were incubated with vehicle control or CDDO-Im (100 nM) for secondary sphere culture as shown in the diagram. Representative microphotographs of secondary tumorspheres after 7-day incubations are shown. Two separate experiments with triplicates were conducted. Averages of the sphere forming efficiency (**p<0.01) with or without CDDO-Im treatment and average number of tumorspheres in each size range are shown in the graph.

To investigate the effects of CDDO-Im on tumorspheres, SUM159 cells were treated with CDDO-Im for 7 days in ultra-low attachment plates. The images of primary tumorspheres of SUM159 cells showed markedly decreased number and size of tumorspheres in cells treated with CDDO-Im (Fig. 4). The sphere forming efficiency (SFE) of SUM159 cells in the primary sphere culture was significantly suppressed by CDDO-Im from 0.46±0.02% to 0.32±0.05% (p<0.01) (Fig. 4). The size of primary tumorspheres was also decreased by CDDO-Im, as evidenced by significantly decreased number of large tumorspheres (>200 µm) by CDDO-Im from 14.7±1.5 to 7.3±2.1 (p<0.01) (Fig. 4). To determine the effect of CDDO-Im on self-renewing capacity to form secondary tumorspheres, SUM159 cells from monolayer culture were first incubated with vehicle control or CDDO-Im for 7 days in sphere culture condition, generating control primary spheres or CDDO-Im primary spheres, respectively. Then, SUM159 cells harvested from primary tumorspheres were treated with vehicle control or CDDO-Im for an additional 7 days to form secondary tumorspheres. The images of secondary tumorspheres of SUM159 cells showed that CDDO-Im treatment markedly decreased the number and size of tumorspheres both from control primary spheres and CDDO-Im primary spheres (Fig. 5). In secondary tumorspheres generated from the control primary spheres, CDDO-Im treatment significantly reduced the SFE (from 1.25±0.08% to 0.96±0.08%, p<0.01) as well as sphere size (Fig. 5). CDDO-Im treatment further decreased the SFE (from 0.89±0.08% to 0.71±0.06%, p<0.01) as well as the size of secondary tumorspheres generated from the CDDO-Im primary spheres (Fig. 5). Notably, the SFE of secondary tumorspheres from the CDDO-Im primary spheres (0.89±0.08%) was lower than those from the control primary spheres (1.25±0.08%) (p<0.01) even without CDDO-Im treatment during the secondary sphere culture (Fig. 5).

### Multiple human stem cell signaling pathways are regulated by CDDO-Im in SUM159 tumorspheres

Many signaling pathways regulating normal stem cells are required for the maintenance of cancer stem cells [13]. Therefore, the effects of CDDO-Im on 84 genes involved in stem cell signaling pathways were evaluated using a human stem cell signaling PCR array. Of 84 genes, 35 of these genes (41.7%) were significantly down-regulated by CDDO-Im in the primary tumorspheres (p<0.05), and those genes are listed in Table 1 (The results for all 84 genes of stem cell signaling tested with PCR array are provided in Table S1). The mRNA levels of Notch receptors, Notch1 (p = 0.015) and Notch3 (p = 0.001), as well as TGF-β superfamily receptors, TGFBR2 (p = 0.004) and TGFBR3 (p<0.001), were significantly decreased by CDDO-Im. The mRNA levels of downstream effectors of Hedgehog signaling, GLI1 (p = 0.038) and GLI3 (p = 0.006), were significantly reduced by CDDO-Im, and this triterpenoid down-regulated the expression of multiple Wnt receptors, including FZD1, FZD4, FZD6, FZD7 and FZD8. However, Wnt signaling was excluded from additional studies because of high PCR cycle numbers, which suggested low expression of these signaling molecules.

**Table 1:** Genes involved in stem cell signaling regulated by CDDO-Im in SUM159 tumorspheres.

Signaling Pathway	Gene ID	Description	Foldchange^a^	p-value^b^
Fibroblast Growth Factor	FGFR1	Fibroblast growth factor receptor 1	0.3	0.001
Hedgehog	GLI1	GLI family zinc finger 1	0.5	0.038
	GLI3	GLI family zinc finger 3	0.5	0.006
	SUFU	Suppressor of fused homolog	0.7	0.003
Notch	NCSTN	Nicastrin	0.7	0.002
	NOTCH1	Notch 1	0.6	0.015
	NOTCH3	Notch 3	0.6	0.001
	PSEN1	Presenilin 1	0.6	0.018
	PSEN2	Presenilin 2	0.6	0.007
TGF-β/Smad	ACVR1	Activin A receptor, type I	0.6	0.006
	ACVR1C	Activin A receptor, type IC	0.4	0.002
	ACVR2B	Activin A receptor, type IIB	0.7	0.023
	BMPR2	Bone morphogenetic protein receptor, type II	0.4	0.001
	ENG	Endoglin	0.7	0.025
	LTBP1	Latent transforming growth factor beta binding protein 1	0.2	0.001
	LTBP2	Latent transforming growth factor beta binding protein 2	0.2	0.001
	LTBP3	Latent transforming growth factor beta binding protein 3	0.3	0.000
	TGFBR2	Transforming growth factor, beta receptor 2	0.6	0.004
	TGFBR3	Transforming growth factor, beta receptor 3	0.7	0.004
	EP300	E1A binding protein p300	0.7	0.002
	SMAD7	SMAD family member 7	0.5	0.009
	SMAD9	SMAD family member 9	0.8	0.009
	CREBBP	CREB binding protein	0.7	0.031
	SP1	Sp1 transcription factor	0.8	0.007
Wnt	FZD1	Frizzled family receptor 1	0.7	0.007
	FZD4	Frizzled family receptor 4	0.6	0.027
	FZD6	Frizzled family receptor 6	0.5	0.008
	FZD7	Frizzled family receptor 7	0.6	0.018
	FZD8	Frizzled family receptor 8	0.3	0.018
	BCL9	B-cell CLL/lymphoma 9	0.5	0.006
	BCL9L	B-cell CLL/lymphoma 9-like	0.6	0.005
	NFAT5	Nuclear factor of activated T-cells 5, tonicity-responsive	0.4	0.001
	PYGO2	Pygopus homolog 2	0.4	0.014
	TCF7L1	Transcription factor 7-like 1	0.7	0.002
	TCF7L2	Transcription factor 7-like 2	0.5	0.004

SUM159 cells were treated with vehicle control or CDDO-Im (100 nM) for 7 days in sphere culture. RNAs were extracted from SUM159 tumorspheres, and the expression level of each gene was analyzed by stem cell signaling PCR array.

a Fold change was determined by the relative fold change of each gene expression level in SUM159 tumorspheres with CDDO-Im treatment as compared to that of SUM159 tumorspheres with vehicle control treatment.

b p-value was determined by student’s *t*-test using two separate experiments in duplicate.

### CDDO-Im regulates Notch, TGF-β/Smad and Hedgehog signaling in SUM159 tumorspheres

**Figure 6:**
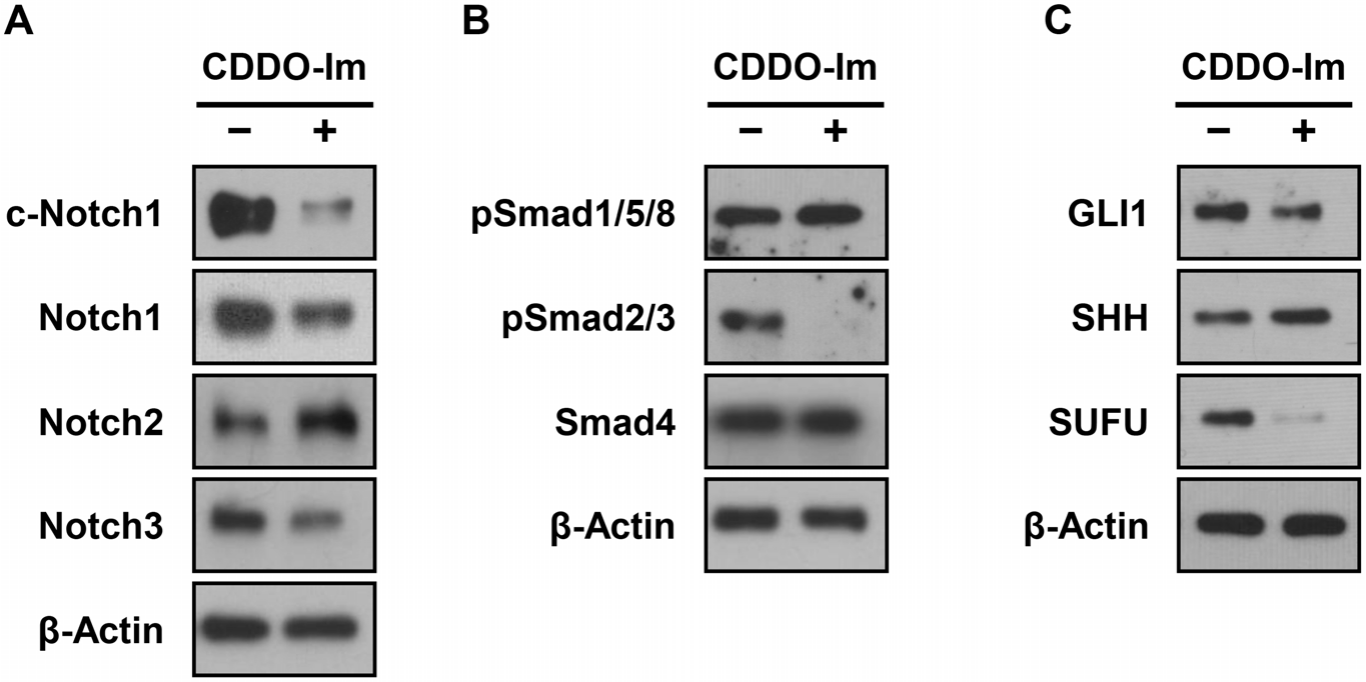
Regulation of the stem cell signaling pathways, Notch, TGF-β/Smad and Hedgehog, by CDDO-Im in SUM159 tumorspheres. SUM159 tumorspheres were incubated with vehicle control or CDDO-Im (100 nM) for 7 days and harvested to investigate stem cell signaling. The protein levels of given molecules for (**A**) Notch, (**B**) TGF-β/Smad and (**C**) Hedgehog signaling pathways in SUM159 cells were determined by Western blot analysis. β-Actin was used as a loading control.

Regulation of Notch, TGF-β/Smad and Hedgehog signaling by CDDO-Im was investigated by measuring the protein levels of key molecules in those signaling pathways. For the Notch signaling pathway, protein levels of the Notch receptors, Notch1, Notch2 and Notch3, were measured. Cleaved Notch1 (c-Notch1), total Notch1 and total Notch3 were markedly repressed by CDDO-Im treatment, while Notch2 was increased (Fig. 6A). For the TGF-β/Smad signaling pathway, the protein levels of downstream effectors, phosphorylated Smad1/5/8 (pSmad1/5/8), phosphorylated Smad2/3 (pSmad2/3) and Smad4 were measured. CDDO-Im markedly inhibited pSmad2/3, but levels of pSmad1/5/8 and SMAD4 were not affected by CDDO-Im (Fig. 6B). For the Hedgehog signaling pathway, the protein levels of GLI1 (a downstream effector), SHH (a ligand) and SUFU (a negative regulator) were determined. GLI1 and SUFU were decreased by CDDO-Im, while SHH showed slight induction by CDDO-Im (Fig. 6C).

## Discussion

In human breast cancer, putative cancer stem cells were first isolated by the expression pattern of cell surface makers, CD44 and CD24, and the breast cancer cells with a CD44^+^/CD24^−/low^ phenotype were able to form tumors in immunodeficient mice with a very low number of cells [4]. Many other markers or phenotypes, such as CD133, CD49f, EpCAM, and aldehyde dehydrogenase activity (ALDH), have also been utilized for the isolation of the breast cancer cells with stem cell-like properties [30]. The majority of cells in many triple-negative breast cancer cell lines were shown to be CD44^+^/CD24^−/low^ cells, and tumorigenic potential was not tightly correlated with the percentage of the CD44^+^/CD24^−/low^ subpopulation [6]. Using EpCAM as an additional cell surface marker, CD44^+^/CD24^−/low^/EpCAM^+^ cells were demonstrated to be a more refined subpopulation with cancer stem cell properties, such as higher tumorigenic potential, self-renewal ability and tumorsphere formation [6]. In the present study, we found that the majority of SUM159 cells were CD44^+^/CD24^−/low^ cells in monolayer, and there was no significant change of those subpopulations in tumorsphere cultures (data not shown). Sphere culture significantly enriched the subpopulation of CD44^+^/CD24^−/low^/EpCAM^+^ cells, supporting the hypothesis that CD44^+^/CD24^−/low^/EpCAM^+^ cells are a more selective cancer stem cell subpopulation than CD44^+^/CD24^−/low^ cells in triple-negative breast cancer. Treatment with CDDO-Im significantly decreased the number of CD44^+^/CD24^−/low^/EpCAM^+^ cells in sphere culture (Fig. 3B), suggesting that CDDO-Im could be used as a potential agent to target cancer stem cells in triple negative breast cancer.

The ability to self-renew is one of the key properties of normal stem cells and cancer stem cells [31]. Suspension sphere cultures have been widely used in stem cell biology to identify and enrich stem cells, as theoretically only stem cells can form spheres with an initial phase of symmetric expansion [32], [33]. In cancer, the ability to form tumorspheres in suspension culture is also used to identify cancer stem cells [31]. Here, we showed that treatment with CDDO-Im significantly reduced the sphere forming efficiency of SUM159 cells (Figs. 4 and 5). The inhibitory effect of CDDO-Im on the sphere forming efficiency was confirmed in other triple-negative and basal-like breast cancer cell lines, SUM149 and MCF10DCIS.com, respectively (Fig. S1). In addition, CDDO-Im markedly decreased the size of tumorspheres, which might reflect the altered proliferation/differentiation status of cancer stem cells or the decreased proliferation of progenitor cells by CDDO-Im (Figs. 4 and 5, and Fig. S1). Interestingly, secondary tumorspheres from the vehicle treated primary tumorspheres showed significantly higher sphere-forming efficiency than the secondary tumorspheres from the CDDO-Im treated primary tumorspheres (Fig. 5). This result might indicate that CDDO-Im inhibits self-renewal of cancer stem cells in primary tumorspheres, causing the decreased cancer stem cells in seeding cells for the successive secondary sphere culture.

In breast cancer, the Notch signaling pathway has been shown to play a critical role in maintaining cancer stem cells by regulating self-renewal [34]. Although most studies have been utilized Notch1 as the readout of Notch signaling, the four Notch receptors, Notch1, Notch2, Notch3 and Notch4, are thought to have different functions in breast cancer [35]. Knockdown of Notch1 or Notch4 inhibited self-renewal and tumorsphere forming ability of breast cancer cells, supporting their roles for the maintenance of cancer stem cells [12], [36]. High activity of Notch3 signaling was associated with aggressive human inflammatory breast cancer and increased lymphovascular invasion, again suggesting tumorigenic activity of Notch3 [37], [38]. On the contrary, high mRNA levels of Notch2 were associated with good clinical outcomes [39]. Moreover, ectopic expression of active Notch2 inhibited cell growth and induced apoptosis in triple-negative breast cancer cells [40], suggesting a tumor suppressor role for Notch2. Interestingly, a recent study demonstrated that withaferin A, a natural chemopreventive agent which is structurally similar to CDDO with a Michael acceptor group, activated Notch2 but inhibited Notch1 activation [41]. In our study, CDDO-Im also induced the protein level of Notch2, while selectively inhibiting Notch1 and Notch3 (Fig. 6A), indicating differential regulation of Notch receptors by CDDO-Im.

Epithelial-mesenchymal transition (EMT) is a cellular process in which adherent epithelial-type cells transform into mesenchymal-type cells [42], and induction of EMT in cancer cells generates cells with stem cell-like properties [43]. TGF-β/Smad signaling is a key regulator of EMT during embryonic development, wound healing and cancer pathogenesis [44]. Moreover, recent studies have demonstrated that activated signaling by the TGF-β superfamily, such as TGF-β, Nodal and Activin, increases the subpopulation of cancer stem cells in breast and pancreatic cancers [45], [46]. CDDO-Im inhibits TGF-β-stimulated cell migration by altering the trafficking and turnover of TGF-β receptors [47]. The present study also demonstrated the inhibitory effect of CDDO-Im on TGF-β/Smad signaling as shown by significantly reduced mRNA levels of Activin and TGF-β receptors (Table 1) as well as decreased protein levels of pSmad2/3 (Fig. 6B). Investigation at to how inhibition of TGF-β signaling by CDDO-Im affects EMT and cancer stem cells in triple negative breast cancer will be an interesting area for future study.

The Hedgehog signaling pathway is involved in patterning and growth of various cells during embryonic development, and its deregulation has been implicated in cancer [13]. The high expression level of GLI1, the key downstream effector of the Hedgehog signaling pathway, is associated with unfavorable overall survival in cancer patients [48], [49]. Moreover, a recent study demonstrated that GLI1 was a central regulator of cancer stem cells in triple-negative breast cancer [50]. In the present study, CDDO-Im decreased both mRNA and protein level of GLI1 in tumorspheres of triple-negative breast cancer (Table 1 and Fig. 6). Since GLI1 is the essential effector of Hedgehog signaling, this result suggests that CDDO-Im inhibits Hedgehog signaling. However, CDDO-Im also reduced SUFU, a negative regulator of Hedgehog signaling, which can activate Hedgehog signaling. Further investigation is necessary in order to understand the direct and feedback mechanisms of CDDO-Im on the Hedgehog signaling pathway.

CDDO and its derivatives, including CDDO-Im, have been shown to induce diverse biological functions in a concentration or cell-type dependent manner [15]. At low concentrations, CDDO-Im (10–100 nM) showed cytoprotective effects by inducing Heme Oxygenase-1 and Nrf2/ARE signaling [51]. On the other hand, high concentrations of CDDO-Im (0.1–1 µM) induced apoptosis in pancreatic cancer cells by rapid depletion of mitochondrial glutathione, causing accumulation of reactive oxygen species [17]. A recent study also showed that high concentrations of CDDO-Im (0.1–1 µM) induced apoptosis in *BRCA*-mutated breast cancer cells by increasing reactive oxygen species (ROS) and DNA damage [19]. However, the same dose of CDDO-Im did not induce ROS in untransformed human breast epithelial cells or mouse fibroblasts [19]. Interestingly, triple-negative breast cancer cells with mutated *BRCA1* or with wild-type *BRCA1* showed high sensitivity to oxidative DNA damage because of their genomic instability and defective DNA-repair system [52]. In the present study, CDDO-Im (100–200 nM) significantly induced apoptosis in SUM159 and MDA-MB-231 cells which are triple-negative breast cancer cells with wild-type *BRCA1* (Fig. 2A and 2B). MDA-MB-231 cells were less sensitive to CDDO-Im than SUM159 cells (Figs. 1B, 1C and 2). This might be because of a gain-of-function p53 mutation in MDA-MB-231 cells, which has been shown to promote cancer cell survival [53]. Overall, our results support CDDO-Im as a potential therapeutic agent to selectively target triple-negative breast cancer cells with genomic instability.

In conclusion, we demonstrated the inhibitory effects of CDDO-Im on triple-negative breast cancer with a potential to target cancer stem cell subpopulation, as evidenced by inhibition of CD44^+^/CD24^−/low^/EpCAM^+^ cells and tumorsphere formation. The present study along with previous reports raises the possibility of using CDDO-Im as a potent anti-cancer agent against triple-negative breast cancer. Since cancer stem cells may be responsible for tumor recurrence and metastasis, the inhibition of multiple stem cell signaling pathways by CDDO-Im might alleviate problems of chemoresistance and metastasis. Because of the concentration-dependent functions of CDDO-Im, preclinical studies to select the proper concentrations to induce anti-cancer activity will be the next step for developing CDDO-Im as a therapeutic agent for triple-negative breast cancer.

## Supporting Information

Figure S1 **Inhibition of sphere forming efficiency and sphere size by CDDO-Im in SUM149 and MCF10DCIS.com cells.** SUM149 (A) and MCF10DCIS.com (B) human breast cancer cells were treated with vehicle control or CDDO-Im (100 nM) for 7 days in sphere culture. Representative microphotographs of tumorspheres after 7-day incubation were shown. Two independent experiments in triplicate were conducted. Averages of the sphere forming efficiency with or without CDDO-Im treatment are shown in the graph (**p<0.01). The size of tumorspheres was divided into three ranges (50∼100, 100∼200 and >200 µm). Average number of tumorspheres in each size range is shown in the graph.
(PDF)

Table S1 **List of 84 stem cell signaling genes in SUM159 tumorspheres and their changes by CDDO-Im.** SUM159 cells were treated with vehicle control or CDDO-Im (100 nM) for 7 days in sphere culture. RNAs were extracted from SUM159 tumorspheres, and the expression level of each gene was analyzed by stem cell signaling PCR array. ^a^: Fold change was determined by the relative fold change of each gene expression level in SUM159 tumorspheres with CDDO-Im treatment as compared to that of SUM159 tumorspheres with vehicle control treatment. ^b^: p-value was determined by student’s *t*-test using two separate experiments in duplicate. ^C^: ND, not detectable.
(PDF)
