# Supplementary figures and images for: A Synthetic Triterpenoid CDDO-Im Inhibits Tumorsphere Formation by Regulating Stem Cell Signaling Pathways in Triple-Negative Breast Cancer
Source: PLoS One. 2014 Sep 17;9(9):e107616. doi: 10.1371/journal.pone.0107616 (PMC4167992; doi:10.1371/journal.pone.0107616)

A

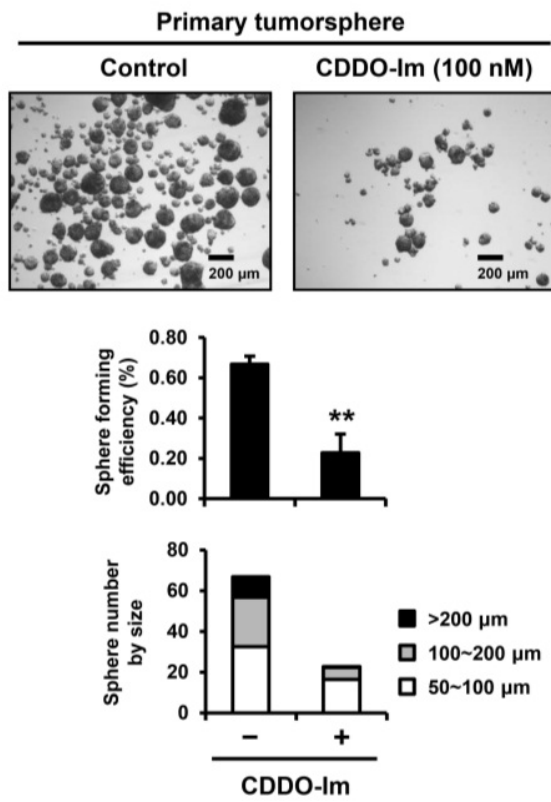

B

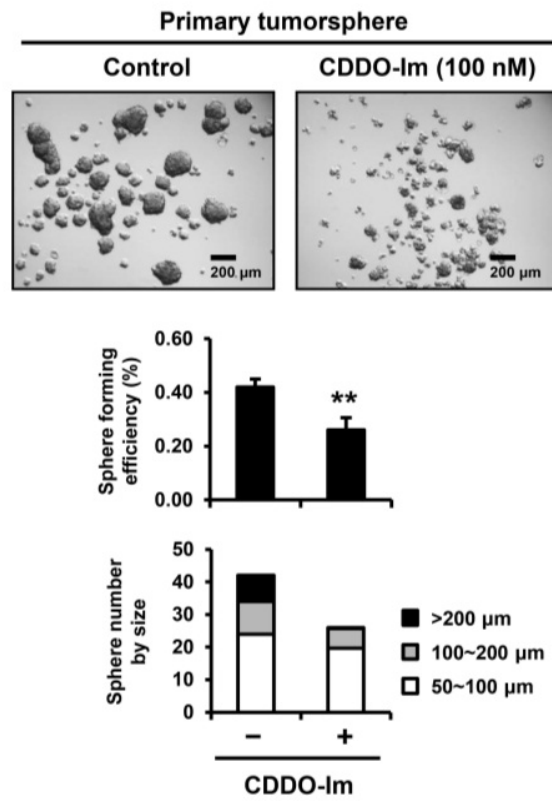

Supplement: Figure S1 — Inhibition of sphere forming efficiency and sphere size by CDDO-Im in SUM149 and MCF10DCIS.com cells. SUM149 (A) and MCF10DCIS.com (B) human breast cancer cells were treated with vehicle control or CDDO-Im (100 nM) for 7 days in sphere culture. Representative microphotographs of tumorspheres after 7-day incubation were shown. Two independent experiments in triplicate were conducted. Averages of the sphere forming efficiency with or without CDDO-Im treatment are shown in the graph (**p<0.01). The size of tumorspheres was divided into three ranges (50∼100, 100∼200 and >200 µm). Average number of tumorspheres in each size range is shown in the graph. (PDF) [file pone.0107616.s001.pdf]
