# Supplementary material for: A Synthetic Triterpenoid CDDO-Im Inhibits Tumorsphere Formation by Regulating Stem Cell Signaling Pathways in Triple-Negative Breast Cancer
Source: PLoS One. 2014 Sep 17;9(9):e107616. doi: 10.1371/journal.pone.0107616 (PMC4167992; doi:10.1371/journal.pone.0107616)
Supplement: Table S1 — List of 84 stem cell signaling genes in SUM159 tumorspheres and their changes by CDDO-Im. SUM159 cells were treated with vehicle control or CDDO-Im (100 nM) for 7 days in sphere culture. RNAs were extracted from SUM159 tumorspheres, and the expression level of each gene was analyzed by stem cell signaling PCR array. a: Fold change was determined by the relative fold change of each gene expression level in SUM159 tumorspheres with CDDO-Im treatment as compared to that of SUM159 tumorspheres with vehicle control treatment. b: p-value was determined by student’s t-test using two separate experiments in duplicate. C: ND, not detectable. (PDF) [file pone.0107616.s002.pdf]

Table S1. List of 84 stem cell signaling genes in SUM159 tumorspheres and their changes by CDDO-Im

Signaling pathway	Gene ID	Fold change[a]	p-value[b]	PCR cycle number	Signaling pathway	Gene ID	Fold change[a]	p-value[b]	PCR cycle number
Pluripotency Maintenance	IL6ST	1.1	0.358	22	Hedgehog	PTCH1	1.2	0.308	32
	LIFR	1.0	0.932	25		PTCHD2	ND	-	>35
	STAT3	1.1	0.691	24		SMO	ND	-	>34
Fibroblast Growth Factor	FGFR1	0.3	<0.001	26		GLI1	0.5	0.038	31
	FGFR2	ND[c]	-	>34		GLI2	ND	-	>33
	FGFR3	ND	-	>33		GLI3	0.5	0.006	26
	FGFR4	ND	-	>33		SUFU	0.7	0.003	29
	CDX2	ND	-	>35	TGF-β/ Smad	ACVRL1	ND	-	>34
Notch	NCSTN	0.7	0.002	26		ACVR1	0.6	0.006	25
	NOTCH1	0.6	0.015	28		ACVR1B	1.1	0.447	31
	NOTCH2	1.0	0.991	23		ACVR1C	0.4	0.002	28
	NOTCH3	0.6	0.001	27		ACVR2A	0.9	0.388	26
	NOTCH4	ND	-	>32		ACVR2B	0.7	0.023	29
	PSENEN	0.7	0.067	26		AMHR2	ND	-	>35
	PSEN1	0.6	0.018	26		BMPR1A	0.8	0.306	25
	PSEN2	0.6	0.007	29		BMPR1B	0.7	0.061	30
	RBPJL	ND	-	>33		BMPR2	0.4	0.001	25
Wnt	FZD1	0.7	0.007	29		ENG	0.7	0.025	27
	FZD2	1.0	0.708	30		LTBP1	0.2	0.000	28
	FZD3	1.1	0.843	26		LTBP2	0.2	0.001	30
	FZD4	0.6	0.027	28		LTBP3	0.3	<0.001	27
	FZD5	1.5	0.054	31		LTBP4	ND	-	>33
	FZD6	0.5	0.008	26		RGMA	ND	-	>37
	FZD7	0.6	0.018	29		TGFBR1	0.8	0.176	26
	FZD8	0.3	0.018	31		TGFBR2	0.6	0.004	24
	FZD9	ND	-	>35		TGFBR3	0.7	0.004	25
	LRP5	0.9	0.319	29		TGFBRAP1	0.9	0.313	27
	LRP6	0.8	0.231	25		EP300	0.7	0.002	27
	VANGL2	ND	-	>32		SMAD1	1.1	0.691	26
	BCL9	0.5	0.006	30		SMAD2	1.0	0.899	25
	BCL9L	0.6	0.005	26		SMAD3	0.9	0.152	30
	CTNNB1	1.0	0.775	25		SMAD4	1.0	0.826	25
	LEF1	0.6	0.063	27		SMAD5	1.0	0.923	24
	NFAT5	0.4	0.001	24		SMAD6	1.0	0.822	31
	NFATC1	ND	-	>32		SMAD7	0.5	0.009	29
	NFATC2	ND	-	>33		SMAD9	0.8	0.009	29
	NFATC3	1.0	0.936	27		CREBBP	0.7	0.031	27
	NFATC4	ND	-	>34		E2F5	0.9	0.787	26
	PYGO2	0.4	0.014	31		RBL1	1.4	0.175	27
	TCF7L1	0.7	0.002	30		RBL2	1.0	0.987	25
	TCF7L2	0.5	0.004	26		SP1	0.8	0.007	26
	TCF7	0.5	0.022	30		ZEB2	1.0	0.698	25
